# Supplementary figures and images for: PPARγ mediated enhanced lipid biogenesis fuels Mycobacterium tuberculosis growth in a drug-tolerant hepatocyte environment
Source: eLife. 2025 Dec 8;14:RP103817. doi: 10.7554/eLife.103817 (PMC12685304; doi:10.7554/eLife.103817)

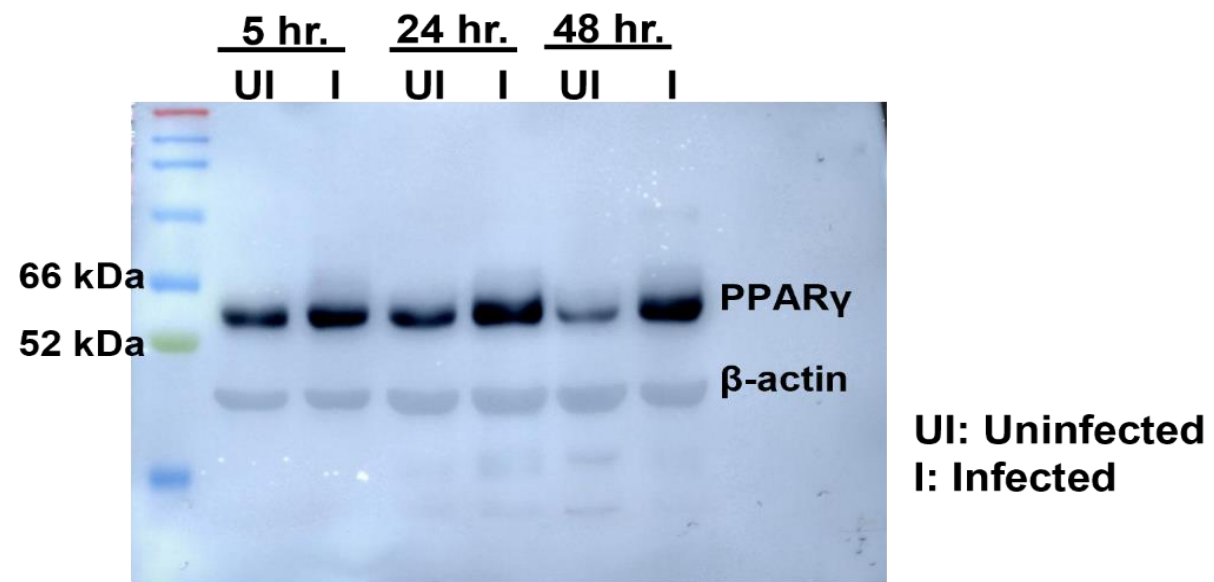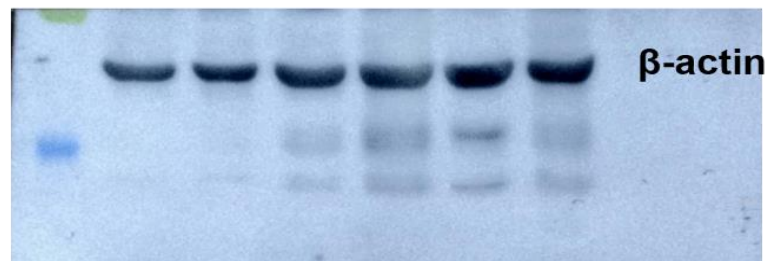

$\beta$ -actin of the same blot, cut and developed separately

Supplement: Figure 6—source data 3. [file elife-103817-fig6-data3.pdf]
