# Supplementary material for: PPARγ mediated enhanced lipid biogenesis fuels Mycobacterium tuberculosis growth in a drug-tolerant hepatocyte environment
Source: eLife. 2025 Dec 8;14:RP103817. doi: 10.7554/eLife.103817 (PMC12685304; doi:10.7554/eLife.103817)
Supplement: Supplementary file 1. [file elife-103817-supp1.docx]

**Details of autopsied liver tissue sections**

1. Case no. 29459/L (Date of Sample registration-10.03.2021) – Age-20y; Sex-M; Diagnosis - ARDS, Disseminated TB.
2. Case no. 29459/10 (Date of Sample registration -10.03.2021) - Age-20y; Sex-M; Diagnosis - ARDS, Disseminated TB
3. Case no. 25901/10 (Date of Sample registration - 10.03.2021) – Age-5y; Sex-F. Diagnosis - TB Meningitis, Stage III communicating hydrocephalus, respiratory failure, increased intracranial pressure, Hyperkalemia, shock.
4. Case no. 26870/9 (Date of Sample registration - 20.04.21) – Age-51y; Sex-M; Diagnosis - Community acquired pneumonia, right exudative pleural effusion, TB with sepsis, refractory septic shock, HIV positive tested outside, Hypertension, acute kidney injury
5. Case no. 8469K (Date of Sample registration - 22.07.21) – case details not available
6. Case no. 27041/6 (Date of Sample registration - 16.01.2019) – Age-66y; Sex-M; Diagnosis - Refractory septic shock, Acute Kidney Injury, CLL, DCMP, AIHA.
